# Why is C_4_ photosynthesis so rare in trees?

**DOI:** 10.1093/jxb/eraa234

**Published:** 2020-05-15

**Authors:** Sophie N R Young, Lawren Sack, Margaret J Sporck-Koehler, Marjorie R Lundgren

**Affiliations:** 1 Lancaster Environment Centre, Lancaster University, Lancaster, UK; 2 Department of Ecology and Evolutionary Biology, University of California, Los Angeles, Los Angeles, CA, USA; 3 Department of Botany, University of Hawaii at Manoa, Honolulu, HI, USA; 4 University of Essex, UK

**Keywords:** C_4_ photosynthesis, *Chamaesyce*, disjunct veins, *Euphorbia*, Euphorbiaceae, phloem loading, symplastic, trees, quantum yield

## Abstract

Since C_4_ photosynthesis was first discovered >50 years ago, researchers have sought to understand how this complex trait evolved from the ancestral C_3_ photosynthetic machinery on >60 occasions. Despite its repeated emergence across the plant kingdom, C_4_ photosynthesis is notably rare in trees, with true C_4_ trees only existing in *Euphorbia*. Here we consider aspects of the C_4_ trait that could limit but not preclude the evolution of a C_4_ tree, including reduced quantum yield, increased energetic demand, reduced adaptive plasticity, evolutionary constraints, and a new theory that the passive symplastic phloem loading mechanism observed in trees, combined with difficulties in maintaining sugar and water transport over a long pathlength, could make C_4_ photosynthesis largely incompatible with the tree lifeform. We conclude that the transition to a tree habit within C_4_ lineages as well as the emergence of C_4_ photosynthesis within pre-existing trees would both face a series of challenges that together explain the global rarity of C_4_ photosynthesis in trees. The C_4_ trees in *Euphorbia* are therefore exceptional in how they have circumvented every potential barrier to the rare C_4_ tree lifeform.

## Teaser

We review possible physiological, anatomical, and evolutionary limitations that may explain the rarity of C_4_ photosynthesis in trees and discuss how the C_4_ trees in *Euphorbia* are exceptions to this.

## Introduction

C_4_ photosynthesis arises from anatomical and biochemical modifications to the ancestral C_3_ photosynthetic machinery that serve to concentrate CO_2_ around Rubisco (Box 1). This CO_2_-concentrating mechanism (CCM) acts to elevate CO_2_ assimilation, while functionally increasing the apparent specificity of Rubisco for CO_2_, over O_2_, thus minimizing oxygenation and resultant photorespiration (Tcherkez *et al.*, 2006). It follows that the evolution of C_4_ photosynthesis is favoured by environmental conditions that would promote high rates of photorespiration in C_3_ species, namely low ambient CO_2_ concentrations, warmth, bright light, aridity, and salinity (Chollet and Ogren, 1975; Ehleringer *et al.*, 1991; Sage *et al.*, 2018). Since C_4_ photosynthesis was first observed >50 years ago (Kortschak *et al.*, 1965; Hatch and Slack, 1966), numerous studies have attempted to elucidate exactly which modifications are typically required to assemble the components of C_4_ physiology (Box 2). To travel the path of C_4_ evolution, an ancestral C_3_ progenitor arrived in an environment selective for a C_4_ benefit (Ehleringer *et al.*, 1997; Sage, 2001*a*; Sage *et al.*, 2018), and, starting from an initial set of genetic and anatomical pre-adaptations (Monson, 2003; Christin *et al*., 2013, 2015; Griffiths *et al.*, 2013), evolved developmental and genetic modifications (Stata *et al.*, 2016; Moreno-Villena *et al.*, 2018; Dunning *et al.*, 2019*a*; Lundgren *et al.*, 2019), navigated energetic constraints (Bellasio and Lundgren, 2016), and underwent progressive optimization (Rondeau *et al.*, 2005; Christin *et al.*, 2009)—or, in some cases, potentially ‘cheated’ this lengthy final step via horizontal gene transfer (Christin *et al*., 2012*a*, *b*; Dunning *et al.*, 2019*b*). Given that some version of this path has been repeatedly travelled nearly 70 times by diverse plant lineages spanning a wide range of lifeforms and ecological niches (Sage *et al.*, 2011*a*), it seems unusual that only a single group of true trees (i.e. defined here as tall, perennial, woody lifeforms with secondary growth) has evolved C_4_ leaves (Pearcy and Troughton, 1975; Table 1). These exceptional trees are members of *Euphorbia*, a global genus of Euphorbiaceae spanning the semideserts of East Africa to the rainforests of the Pacific Islands, and encompassing growth forms from herbs to xerophytic stem succulents to trees of up to 30 m in height (Horn *et al.*, 2012).

**Box 1. C_4_ photosynthesis arises from both anatomical and biochemical modifications to the ancestral C_3_ photosynthetic system**

There are several anatomical and biochemical differences that arise during the transition from the C_3_ photosynthetic system to the C_4_ CO_2_-concentrating mechanism (CCM). In C_3_ plants, the majority of chloroplasts (and associated Rubisco) are localized to the mesophyll, which is largely exposed to ambient CO_2_ and O_2_ concentrations. The featureless nature of CO_2_ and O_2_ makes them enzymatically hard to distinguish, such that Rubisco has catalytic affinity for both molecules, and will catalyse the carboxylation and oxygenation of RuBP. Because the oxygen availability to Rubisco is high in C_3_ plants, oxygenation and subsequently photorespiration occur at high rates, especially in high-temperature and low-CO_2_ conditions. Some C_3_ plants have evolved higher specificity of Rubisco for CO_2_ over O_2_; however, this comes at the cost of slower catalytic turnover (Tcherkez *et al.*, 2006).

The C_4_ CCM allows plants to avoid this specificity–efficiency trade-off by increasing CO_2_ concentrations at the Rubisco active site, leading to an apparent increase in the specificity of Rubisco for CO_2_. In C_4_ plants, CO_2_ is biochemically shuttled from the mesophyll into the bundle sheath via the carbonic anhydrase–phosphoenolpyruvate carboxylase (PEPC) system, which builds up CO_2_ concentrations around Rubisco within bundle sheath cells, drastically reducing the incidence of oxygenation and increasing net carbon assimilation.

To facilitate the C_4_ cycle, C_4_ plants have higher bundle sheath to mesophyll area ratios (Hattersley, 1984), often via increased density of vascular bundles (Lundgren *et al.*, 2019). In addition, the connectivity of the mesophyll and bundle sheath cells is enhanced in C_4_ plants by an increased density of plasmodesmata at the cell interface, which allows for the increased flux of metabolites that is required for a functional C_4_ cycle (Danila *et al.*, 2016).

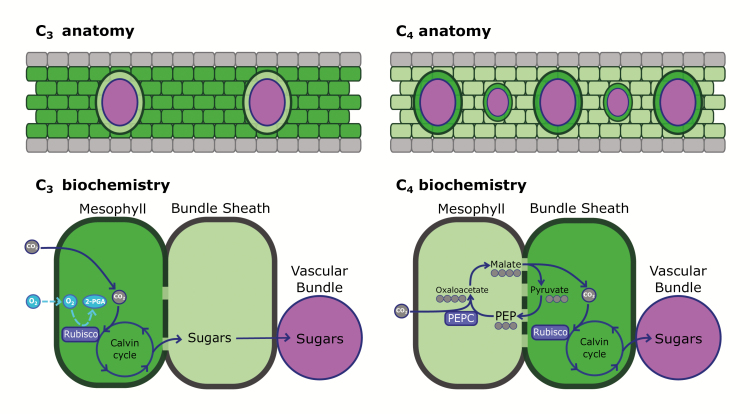

Grey, leaf epidermis; dark green, cells with many chloroplasts; light green, cells with fewer chloroplasts; purple circles/ovals, vascular tissue; solid purple lines, photosynthesis; dashed light blue lines, oxygenation; dashed dark green line, mesophyll–bundle sheath interface.

**Box 2. Key developments in understanding C_4_ evolution**

There are numerous evolutionary steps on the path from C_3_ to C_4_ (Stata *et al.*, 2019)_._ To understand this complex evolutionary progression, it is most useful to examine it in study systems that contain individuals across the entire spectrum of photosynthetic types from C_3_ to C_4_. While there are many fully C_3_ and C_4_*Euphorbia* species, there are only two known C_3_–C_4_ intermediates, and this limits the use of *Euphorbia* as a model for understanding C_4_ evolution. However, study of the eudicot genus *Flaveria* has been crucial in understanding the evolutionary transitions from C_3_–C_4_ to C_4_ in particular (Monson and Moore, 1989), while studies of the grass *Alloteropsis semialata* provide unparalleled insight into C_4_ evolution owing to its extreme intraspecific photosynthetic diversity, which reduces the confounding effects of long divergence times in phylogenetic analyses and species comparisons (Lundgren *et al.*, 2016; Dunning *et al.*, 2019*a*). Key developments in these systems have started to pick apart the anatomical and biochemical components required to construct a functional C_4_ photosynthetic system. The remarkable convergence of the C_4_ trait across the plant kingdom means that the findings from *Flaveria* and *A. semialata* can be applied to other distantly related C_4_ lineages, such as *Euphorbia,* despite differences in life history, to understand which stages on the path to C_4_ might conflict with the tree habit.

- ** High expression of key genes is important in the establishment of a weak C**_**4**_ **cycle.** Moreno-Villena *et al.* (2018) demonstrated that highly expressed genes in grasses, and also possibly in *Flaveria*, were preferentially co-opted for C_4_ photosynthesis regardless of tissue specificity. The importance of high expression levels of C_4_ cycle genes was further shown by the observed increases in expression of the key C_4_ genes phosphenolpyruvate carboxykinase (PEPCK) and phosphenolpyruvate carboxylase (PEPC) across the C_3_ to C_3_–C_4_ transition (Dunning *et al.*, 2019*a*). These genes underwent duplication and a resultant dosage-dependent increase concurrently with their co-option for C_4_ photosynthesis in *Alloteropsis semialata* (Bianconi *et al.*, 2018).
- ** C**_**4**_ **anatomy can evolve via a single developmental change: an increase in vein density.** Lundgren *et al.* (2019) demonstrated that an increase in vein density driven by proliferation of minor veins is sufficient, given necessary anatomical pre-conditions, to produce a functional C_4_ leaf anatomy and create an evolutionary entry to more complex C_4_ syndromes in *A. semialata*. Christin *et al.* (2013) present the necessary anatomical pre-conditions (or ‘enablers’) in grasses as a high (>15%) proportion of bundle sheath tissue (combination of a short distance between bundle sheaths and large bundle sheath cells), which, when combined with environmental changes, facilitated the emergence of the C_4_ trait.
- ** A reduction in chloroplast numbers and increased chloroplast size is associated with changes in C**_**4**_ **metabolic activity.** Stata *et al.* (2016) showed that across C_3_, C_3_–C_4_, and C_4_ species in the genus *Flaveria*, chloroplast number and coverage of the mesophyll cell periphery increase with increased strength of C_4_ metabolism, while increased C_4_ cycle strength was associated with increased chloroplast size. The reduced chloroplast volume at the mesophyll cell periphery and associated increased cytosolic exposure to the atmosphere could enhance diffusion of CO_2_ to PEPC, thus facilitating the incorporation of CO_2_ into the C_4_ metabolic cycle.
- ** Lateral gene transfer is an important force in C**_**4**_ **evolution, spreading functional genes among grasses**. A total of 59 genes in the genome of *A. semialata* were laterally acquired from other grasses, including those known to be involved in C_4_ photosynthesis (Dunning *et al.*, 2019*b*; Olofsson *et al.*, 2019).

While these anatomical and biochemical modifications that assemble the C_4_ trait were selected for by increased fitness in hot, dry environments, they have been retained by the entire Hawaiian *Euphorbia* across multiple transitions from open habitats into the moist forest understorey following the colonization of the Hawaiian Islands, and multiple transitions to the tree habit (Yang *et al.*, 2018). Considering these recent developments in our understanding of C_4_ evolution, alongside the ecology and biogeography of the Hawaiian radiation, is key to understanding the overall evolutionary progression of the lineage.

**Table 1.** Where are C_4_ trees found?

Species	Varieties	Form	Geography	Environment	Reference
Euphorbiaceae					
*Euphorbia olowaluana*		Tree (up to 9 m)	Open and subalpine forest	Dry	a, b, d, e, f
E. herbstii*		Tree (3–8 m)	Forest	Mesic to wet	a, c, d, e, f
*E. remyi*	*kauaiensis*	Small tree (2–3 m)	Forest^b,d,e^	Wet ^b,d,e^	a, b, d, e, f
*E. rockii*	*rockii* ^b,e^ *grandifolia* ^b,e^	Shrub to tree (1–8 m)	Open ridge to forest	Mesic to wet	a, b, d, e, f
*E. celastroides*	*lorifolia*	Shrub to tree (1–9 m)	Open forest	Dry	a, b, d, e, f
*E. atrococca*		Shrub to small tree (up to 3 m)	Forest	Dry to mesic	a, b, d, e, f
Chenopodiaceae (tribe Salsoleae *sensu stricto*)					
Haloxylon persicum**		Large shrub to tree (up to 8 m)	Desert	Dry	g, h, i
H. ammodendron**		Large shrub to tree (up to 8 m)	Desert	Dry	g, h, i

^a^ Pearcy and Troughton, 1975; ^b^Koutnik, 1987; ^c^Robichaux and Pearcy, 1980; ^d^Sporck, 2011 (p70); ^e^Yang *et al.*, 2018; ^f^Yang, 2012; ^g^Sage, 2001*a*, *b*; ^h^Sage, 2016; ^i^Pyankov *et al.*, 1999.

*formerly *E. forbesii*; **these species have C_4_ photosynthetic stems, C_3_ leaf-like cotyledons, and no true leaves, and become arborescent with age.

*Euphorbia* is the only known genus to contain plants using crassulacean acid metabolism (CAM), C_3_, C_3_–C_4_, and C_4_ photosynthetic types (Yang and Berry, 2011). The C_4_ lineage in *Euphorbia*, the largest single C_4_ lineage among the eudicots, is found within subgenus *Chamaesyce*, a subclade of *Euphorbia* that includes C_3_ and C_4_ species, as well as C_3_–C_4_ evolutionary intermediates (Sage *et al*., 1999*a*, 2011*b*; Yang and Berry, 2011; Box 3). Subgenus *Chamaesyce* underwent a radiation on the Hawaiian Islands, resulting in 27 taxa that all use C_4_ photosynthesis, as indicated by δ ^13^C values spanning –14.5‰ to –12.0 ‰ (Pearcy and Troughton, 1975; Sporck, 2011). The Hawaiian species in *Chamaesyce* (hereafter referred to as Hawaiian *Euphorbia*, still recognizing that a separate colonization event led to the origin of a C_3_ Hawaiian tree not in *Chamaesyce*, *Euphorbia haeleeleana*) include a variety of growth forms, from sub-shrub to tree, which exist across a diverse range of environments, from bright and arid habitats to mesic and wet forest understoreys (Table 1). Though there are some relatively shade-tolerant C_4_ monocots (e.g. Lundgren *et al.*, 2015), shade-tolerant C_4_ eudicots are rare (Sage *et al.*, 1999*b*) and thus the ubiquity of C_4_ photosynthesis across the diverse habitats of Hawaiian *Euphorbia* is surprising (Pearcy and Troughton, 1975; Sage, 2016). Given that the entire Hawaiian radiation uses C_4_ photosynthesis, it is likely that the progenitor for this radiation was also a C_4_ species that arrived on the Hawaiian Islands. This idea is supported by recent phylogenetic work that suggests that the closest relatives of Hawaiian *Euphorbia* are in fact C_4_ herbs from the southern USA, Mexico, and/or the Caribbean, and that the woody state evolved after arrival on the Hawaiian Islands (Yang and Berry, 2011; Yang *et al.*, 2018; Box 3).

**Box 3. Phylogenetic relationships in *Euphorbia* and closest relatives of Hawaiian taxa**

C_4_ photosynthesis is present in all species of the core *Chamaesyce*, which includes both the Hypercifolia and Peplis clades. A phylogeny of the *Chamaesyce* clade (*Euphorbia* subg. *Chamaesyce* sect. *Anisophyllum*) identifies four close relatives and possible progenitors of Hawaiian *Euphorbia*: *Euphorbia stictospora*, *E. velleriflora*, *E. mendezii*, and *E. leucantha* (Yang and Berry, 2011). These species are members of the Hypercifolia clade and are herbaceous annuals commonly found in the southern USA, northern Mexico, and/or the Caribbean. *Euphorbia haeleeleana*, a woody C_3_ species that is part of *Euphorbia* subg. *Euphorbia*, represents a separate colonization of the Hawaiian Islands. The closest relatives of this taxa are the Australian succulents *E. plumerioides* and *E. sarcostemmoides*, also from subg. *Euphorbia* (Zimmermann *et al.*, 2010).

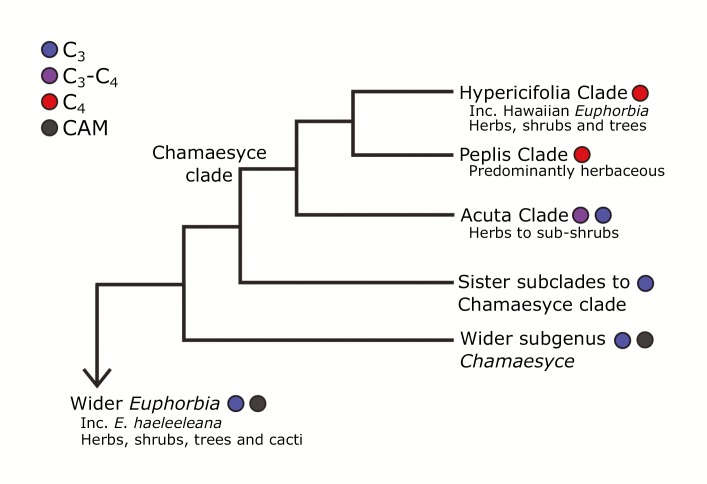

There has been periodic interest in Hawaiian *Euphorbia*, and the wider rarity of C_4_ trees (e.g. Pearcy and Troughton, 1975; Ehleringer, 1978; Pearcy, 1983; Sporck, 2011; Sage, 2014; Sage and Sultmanis, 2016; Yang *et al.*, 2018); however, there has been little definitive progress towards understanding why C_4_ trees are indeed so rare. Given that C_4_ trees do exist, there cannot be any fundamental incompatibility between C_4_ photosynthesis and the tree habit, or any physiological explanation for the reduced competitive ability of a C_4_ tree versus a C_3_ tree that is true under all conditions. Therefore, in explaining the rarity of C_4_ trees, all the factors that could limit, but not preclude, the evolution of the two syndromes in the same species must be considered, whether that evolution is via adaptation of a C_4_ progenitor to an environment inhabited by trees, as is likely to be the case for Hawaiian *Euphorbia*, or via an existing tree traversing the adaptive landscape from C_3_ to C_3_–C_4_ to C_4_ (Yang and Berry, 2011). This review will consider the key steps on the path to C_4_ photosynthesis, where these steps might conflict with the tree lifeform, and argue that Hawaiian *Euphorbia* present a unique opportunity to study the evolution of the C_4_ trait in trees as a target for future research.

## C_4_ trees may perform poorly under a closed canopy

Rates of photorespiration increase in warm environments, making the C_4_ pathway—which largely avoids photorespiration—superior to C_3_ photosynthesis in plants with similar lifeforms (Ehleringer, 1978). As such, C_4_ grasses frequently dominate in areas with warm climates where trees cannot grow, for example due to high levels of disturbance, while C_3_ forests—and thus canopies—establish in warm areas where conditions are such that trees thrive. The theory that follows is that C_4_ trees have failed to become widespread forest species due to their poor performance under canopies, where conditions are cool, shady, and often enriched in CO_2_ (Sage *et al.*, 1999*a*). However, this theory may not hold true given insights into the physiological performance of *Euphorbia* species in the Hawaiian forest understorey (Robichaux and Pearcy, 1980; Pearcy *et al.*, 1985) and, more recently, C_4_ photosynthesis under low light canopy conditions (Bellasio and Griffiths, 2014; Sage, 2014).

### Reduced quantum yield as a limitation

Ehleringer (1978) proposed an early hypothesis that the quantum yield of photosynthesis, defined as the rate of photosynthesis relative to that of photon absorption, is important in determining the distribution of C_4_ species, especially grasses. Maximum quantum yield is inherently lower in C_4_ plants than in C_3_ plants due to the greater energy requirements of the C_4_ system, though the quantum yield of C_3_ plants declines with increasing temperature while that of C_4_ plants remains constant (Long, 1999; Monson, 1999). If below-canopy temperatures are sufficiently cool that the additional energy requirement of the C_4_ system is greater than the light energy lost to photorespiration in an energetically inexpensive C_3_ plant, then the quantum yield of the C_3_ plant would be greater than that of a theoretical C_4_ tree, and that tree would probably be outcompeted. Indeed, for *Atriplex* species native to grassland, desert, and coastal strand habitats, the temperature at which the quantum yields of C_3_ and C_4_ species are equal is 30 °C, at least at atmospheric CO_2_ (at that time 325 ppm) and O_2_ concentrations (Ehleringer and Björkman, 1977). Quantum yield also varies with CO_2_ concentration, life history, and C_4_ biochemical subtype. The lower quantum yield of eudicots compared with monocots may contribute to the relative scarcity of shade-tolerant C_4_ eudicot herbs compared with forest-shade grasses (Monson, 1999). Similarly, the higher quantum yields of plants using the NADP-ME biochemical subtype of C_4_ photosynthesis (such as Hawaiian *Euphorbia*) compared with those using the NAD-ME subtype (Ehleringer and Pearcy, 1983; Pearcy and Ehleringer, 1984) may partially explain the shade tolerance of the understorey C_4_ tree *Euphorbia herbstii* (formerly *E. forbesii*), whose quantum yield equals that of an equivalent C_3_ tree at a leaf temperature of 22–23 °C (approximately the same value as the mean midday leaf temperature at the site where these plants were collected) (Robichaux and Pearcy, 1980).

Direct comparison between the quantum yields of C_3_ and C_4_ species, however, does not adequately address the question of whether or not quantum yield could limit the evolution of a C_4_ tree: the quantum yields of intermediate species on the path from C_3_ to C_4_ must be considered. In the incipient C_3_–C_4_ phases of C_4_ evolution in *Flaveria*, the poor integration of the C_3_ and C_4_ cycles causes futile cycling in the C_4_ assimilation of CO_2_ and thus reduced quantum yields (Monson *et al.*, 1986; Stata *et al.*, 2019). Inefficient transfer of CO_2_ from the C_4_ to the C_3_ cycle may create an ‘adaptive trough’, expressed through reduced quantum yields in C_3_–C_4_ species, compared with fully coupled C_3_ or C_4_ taxa, which could act as a barrier to the evolution of C_3_–C_4_ traits in species native to shady habitats (Monson, 1989). Thus, the limitations of the C_3_–C_4_ intermediate state could make it difficult for the C_4_ pathway to evolve in a tree under a forest canopy. However, this limitation does not apply where the transition to the forest understorey occurred subsequently to the evolution of the full C_4_ trait, as was likely to have been the case for Hawaiian *Euphorbia* (Yang and Berry, 2011).

### Poor ability to utilize sunflecks as a limitation

Sage *et al.* (1999*a*) proposed that C_4_ plants may be maladapted to shady understorey environments due to their inefficient utilization of sunflecks, which represent the primary source of light available under the canopy. However, sunfleck use does not seem to be a limiting factor in *Euphorbia*, as the C_4_ tree *E. herbstii* is as efficient in utilizing sunflecks as a comparative C_3_ tree (Pearcy *et al.*, 1985). Similarly, study of the C_4_ grass maize shows that, while it responds more slowly to short sunflecks, it otherwise has a similar sunfleck use efficiency to C_3_ crop species (Krall and Pearcy, 1993), suggesting that C_4_ plants may not be inherently limited by poor sunfleck exploitation.

### Inability to meet the increased energy demands of C_4_ in the shade

Under low light, C_4_ plants suffer increased ‘bundle sheath leakiness’—the rate of diffusion of CO_2_ out of the bundle sheath relative to that of phosphoenolpyruyvate (PEP) carboxylation (Kromdijk *et al.*, 2008). This is driven by a slow down in the C_4_ carboxylation process due to a reduction in ATP availability under low light. Thus, in the low-light forest interior, the higher leakiness would limit carbon gain and lead to poorer performance of a theoretical C_4_ tree. However, maize plants grown under diffuse light can acclimate and thereby reduce leakiness, possibly by allocating proportionally more energy to C_3_ cycle activity to reduce CO_2_ overcycling, optimizing scarce ATP resources, and then trapping a greater proportion of CO_2_ in the bundle sheath (Bellasio and Griffiths, 2014).

Disjunct veins, which shade-tolerant Hawaiian *Euphorbia* species possess (Herbst, 1971), may be another mechanism adapted to tolerate shade. This modification to leaf anatomy allows these species to have a low density of functional veins (i.e. those that are connected to the vascular network) to reduce leaf costs, as is typical of shade species (Sporck, 2011), while establishing islands of bundle sheath tissue to increase the relative bundle sheath tissue area and maintain the close proximity of mesophyll and bundle sheath cells required for a functional C_4_ system (Box 1). Therefore, it seems that optimization of C_4_ physiology in combination with modifications to leaf anatomy should allow C_4_ plants to thrive under the canopy, including C_4_ trees such as *E. herbstii* and *E. rockii* (Table 1). However, not all C_4_ lineages may be equally primed to adopt these modifications.

## Evolutionary factors shaped the pathway to C_4_ trees

There are several key historical factors and evolutionary ‘opportunities’ that have played a role in shaping the evolution of the C_4_ trait, and subsequently the tree habit, in Hawaiian *Euphorbia*.

### Evolution of the C_4_ trait in *Euphorbia*

First, it is important to consider the age of the eudicot C_4_ lineages and the timing of C_4_ evolution relative to historical climatic changes. C_4_ eudicots are not overall younger than C_4_ monocots, and all lineages of C_4_ plants evolved in the low CO_2_ atmosphere that has shaped plant evolution over the last 30 million years (Christin *et al.*, 2011). This low CO_2_ atmosphere was probably a key evolutionary opportunity for *Euphorbia* and is associated with the evolution of at least 17 independent CCMs, which are mostly CAM but also include the C_4_ lineage (subsect. Hypericifoliae) of *Euphorbia* subg. *Chamaesyce* (Horn *et al.*, 2014; Sage, 2016).

Secondly, relatively short generation times in *Euphorbia*, owing to rapid flowering and high levels of reproductive output per plant, favour the comparatively fast evolution of traits, probably including those associated with C_4_ photosynthesis, as well as the rapid accumulation of duplicated and neofunctionalized genes as a resource for C_4_ evolution (Monson, 2003; Emms *et al.*, 2016; Bianconi *et al.*, 2018; Box 2). However, this rapid generation time probably offers less potential evolutionary benefit than lateral gene transfer, which is increasingly recognized as an important force in shaping C_4_ evolution in the monocots, but has not been documented in C_4_ eudicots (Christin *et al*., 2012*a*, *b*; Dunning *et al.*, 2019*b*; Olofsson *et al.*, 2019; Box 2).

Thirdly, lifeform may have also played a role in the evolutionary potential of these plants. The ancestor of the C_4_ lineage in *Euphorbia* was likely to have been herbaceous, while all 16–21 independent origins of CAM in *Euphorbia* occurred in woody ancestors (Horn *et al.*, 2014). While this is only a single example, it suggests that the woody ancestor of *Euphorbia* may have needed to undergo a transition to the herbaceous lifeform prior to the evolution to C_4_ photosynthesis, and perhaps the evolution of C_4_ is less favourable than that of CAM in a woody species. Furthermore, the herbaceous lifeform of the ancestor of Hawaiian *Euphorbia* facilitated its dispersal to the Hawaiian Islands (Yang and Berry, 2011). Indeed, much of the Hawaiian woody flora evolved from herbaceous ancestors, which had greater dispersal ability to reach the remote Hawaiian Islands (Carlquist, 1970; Panero *et al.*, 1999; Eggens *et al.*, 2007; Dunbar-Co *et al.*, 2008). Before European contact and the influx of invasive species, Hawaii had an environment with available niches along with topographic heterogeneity providing barriers to gene flow (Givnish *et al.*, 2009). Thus, C_4_ trees or their progenitors (none of which are able to dominate the forest canopy) did not need to invade established C_3_ communities or areas of high disturbance that typically occlude the tree lifeform, so providing an evolutionary opportunity for a C_4_ ancestor to transition to the tree habit.

Finally, *Euphorbia* have a remarkably high degree of variation in morphological characteristics, level of adaptive plasticity, and species richness compared with other plant lineages of similar age (Horn *et al.*, 2012). In particular, there are several characteristics of the ancestral *Euphorbia* that may be synergistically associated with the origin of the C_4_ lineage, and thus may have facilitated C_4_ evolution. The combination of plagiotropic branches and a distichous leaf arrangement maximize the leaf area exposed to sunlight. The co-evolution of C_4_ photosynthesis with these growth traits would maximize photosynthetic rates and minimize photorespiration rates in high-light, high-temperature environments. In addition, the high adaptive plasticity of *Euphorbia* may have facilitated the evolution of further adaptations in progenitors of the C_4_ trees, for example the development of shade-tolerant leaves, circumventing constraints that the C_4_ state places on phenotypic plasticity (Herbst, 1971; Robichaux and Pearcy, 1980; Sage and McKown, 2006; Horn *et al.*, 2012; Lundgren *et al.*, 2014). It is also worth noting that adaptive plasticity in Hawaiian *Euphorbia* specifically may have been furthered by their allopolyploid origin resulting in increased heterozygosity (Yang and Berry, 2011).

### Evolution of the tree habit in C_4_*Euphorbia*

There are many factors that drive, and constrain, the evolution of the tree habit. These include, but are not limited to, protection from animal herbivory, improved dispersal, avoiding (self-)shading, and maintaining water balance. It is unclear what has driven the evolution of trees in *Chamaesyce*, as Hawaiian *Euphorbia* tree taxa do not appear to perform better than closely related shrub taxa by measure of abundance.

In terms of evolutionary constraints, in order to diversify to a forest understorey niche, such as that of *E. herbstii*, a C_4_ tree must acquire some shade tolerance. However, shade tolerance does not universally constrain the evolution of the tree habit: C_4_ shrubs can displace grasses in high-light scrubland, as is observed for the C_4_ shrubs *Atriplex confertifolia* and *A. canescens* (Sage and Sultmanis, 2016), and the C_4_ tree *Euphorbia olowaluana* is a pioneer species on newly formed Hawaiian lava fields, occurring sparsely in high-light conditions (see fig. 1H in Yang and Berry, 2011). Therefore, the ability to develop shade-tolerant leaves alone does not dictate whether or not a C_4_ plant can evolve the tree habit, and there may be other factors acting to constrain the evolution of trees in existing C_4_ lineages, such as the age of these lineages (Sage and Sultmanis, 2016).

The evolution of the tree habit in a herbaceous C_4_ ancestor requires sufficient evolutionary time following the appearance of the C_4_ trait. C_4_ trees, and C_4_ shrubs that become arborescent with age, are found in two of the oldest C_4_ eudicot lineages: *Euphorbia* (19.3 Mya) and tribe Salsoleae *sensu stricto* (Chenopodiaceae, 23.4 Mya), respectively (Table 1; Sage and Sultmanis, 2016). However, in the case of Hawaiian *Euphorbia*, 19.3 My is much longer than the time required for the transition from herbaceous to tree lifeform: the initial colonization event of the Hawaiian Islands was ~5 Mya and the true tree species themselves are ~1 My old (Yang *et al.*, 2018). Therefore, it may be more accurate to say that it is not evolutionary time, but the rate of evolution that can act to constrain the transition to a tree lifeform in a C_4_ lineage. Many eudicot families that have C_4_ lineages also have trees, but it may be that the C_4_ state limits the rate at which the tree lifeform can be acquired within C_4_ lineages by reducing adaptive plasticity (Sage and McKown, 2006; Bellasio and Lundgren, 2016). The aforementioned high adaptive plasticity of Hawaiian *Euphorbia*, or the fact that the lineage had woody ancestors that had previously undergone a transition to the herbaceous state, may have favoured a comparatively rapid evolution of the tree lifeform in this C_4_ lineage (Horn *et al.*, 2014). Interestingly, the ancestor of the C_4_ lineage in Salsoleae may have been a shrub or sub-shrub (Schüssler *et al.*, 2017), so while the transition to true tree has not been completed and thus is slower than that in *Euphorbia*, they may have been advantaged in this transition by acquiring C_4_ photosynthesis in an already woody or semi-woody ancestor.

### Passive symplastic phloem loading and/or hydraulic limitation negate the benefits of C_4_ anatomy in trees

Tree height, and indeed the tree growth form, is limited by difficulties in sustaining water and sugar transport over the long pathlength (Ryan and Asao, 2014; Liesche *et al.*, 2017; Savage *et al.*, 2017). We propose that three elements of sugar and water transport design can contribute to limitations on the evolution of C_4_ trees.

First, trees tend to exhibit high numbers of plasmodesmatal connections between mesophyll cells and minor vein cells that are devoted to phloem loading, a phenotype that is frequently associated with a passive symplastic loading mechanism (Davidson *et al.*, 2011). The persistently strong sugar sinks of trees, which have meristems and storage sites in their trunks and roots, and also high rates of photosynthesis and thus photosynthetic export from leaves, may actually select for passive symplastic phloem loading as it is less energetically demanding than active mechanisms (Turgeon, 2010). C_4_ species, on the other hand, have a high density of plasmodesmatal contacts between mesophyll and bundle sheath cells to allow for the flux of intermediate metabolites (Sowiński *et al.*, 2008; Danila *et al.*, 2016). In a C_4_ passive phloem loader, the export of photosynthate would require a large number of plasmodesmatal contacts between bundle sheath and phloem cells. However, this would form a complete plasmodesmatal route from mesophyll to phloem, with C_4_ intermediates moving from mesophyll to bundle sheath (and back), while sugar loading proceeds from the bundle sheath into the phloem. Due to the passive nature of this process and lack of compartmentalization, it would be difficult to regulate the flux of C_4_ metabolites; that is, prevent leakage of C_4_ intermediates into the phloem. Avoiding leakage of C_4_ sugars may place a limit on phloem loading via plasmodesmata and thus on passive loading.

Secondly, the combination of C_4_ intermediate diffusion with direct plasmodesmatal pathways for sugar transport from the mesophyll to the bundle sheath and into the phloem might be very difficult to sustain, given that sugar movement would be against the transpiration stream. Theoretical analysis has shown that transpiration-induced bulk flow from veins to stomata and passive sugar loading into the phloem by diffusion can co-exist (Rockwell *et al.*, 2018), but these analyses assumed very low plasmodesmatal fluxes between the bundle sheath and mesophyll, as is typical of C_3_ species (i.e. without the extensive plasmodesmatal contact typical of C_4_ species). More work is needed to determine if transpirational counterflow might present an obstacle to a C_4_ passive loader.

Thirdly, taller trees tend to have upper canopy leaves with both lower leaf water potentials and a greater heterogeneity in cell water potentials, due to the tension associated with gravity, greater resistance pathlengths, and exposure of canopies to strong fluctuations in light and temperature (Zwieniecki *et al.*, 2004; Burgess and Dawson, 2007). Plasmodesmata may lose transport capacity when strong pressure differences are generated between adjacent cells or tissues (Oparka and Prior, 1992). Thus, the C_4_ pathway, depending on plasmodesmatal transport, may not be feasible at very negative leaf water potentials and/or given the large heterogeneity of water status within the leaf, and so its evolution may be precluded in trees, particularly tall canopy-forming species unlike Hawaiian *Euphorbia* (Table 1).

Even if these three limitations could be overcome, the increased photosynthetic efficiency of the C_4_ system combined with the reduced ratio of source tissue to phloem requires an increase in phloem loading efficiency to avoid accumulation of photosynthate in the leaves. In C_4_ grasses, this is achieved by two mechanisms: first, by up-regulation of active transporters for bundle sheath sugar export, such as maize SWEET-13, a transporter that was duplicated and retained during C_4_ evolution (Emms *et al.*, 2016); and secondly, by increasing plasmodesmatal density at the interface of the bundle sheath and the vascular parenchyma to increase passive photosynthate transport in plants grown under high-light conditions (Sowiński *et al.*, 2007). Such adjustments would probably not be possible or effective in a C_4_ tree, in the first instance owing to the absence of an active loading mechanism, and in the second instance due to the aforementioned limitations on plasmodesmatal transport. Inability to increase the maximum rate of phloem loading and associated accumulation of leaf non-structural carbohydrates would result in a downward adjustment of photosynthetic capacity that could partially or fully negate the potential benefits of the C_4_ system in a tree (Paul and Foyer, 2001; Paul and Pellny, 2003).

How the trees of Hawaiian *Euphorbia* have circumvented these limitations is unknown, and little is known of their phloem loading mechanism. Notably, the anatomical diversity of Hawaiian *Euphorbia* is exceptional, from their striking disjunct veins, which vary strongly across species, to their variation in growth form, leaf surfaces, and leaf cross-sectional anatomy (Herbst, 1971; Koutnik, 1987; Horn *et al.*, 2012). More work is needed to discover how phloem loading relates to this diversity, and if a specialized anatomy evolved that mitigates limitations on C_4_ tree evolution dictated by phloem processes.

## Conclusions

Previous commentary on the rarity of C_4_ trees has pointed towards hypotheses of physiology and life history, and found that there is no single explanation that is satisfactory, with Hawaiian *Euphorbia* (and possibly Salsoleae *sensu stricto*, Table 1) acting as exceptions to every argument. Each hypothesis seems to have a caveat, whereby if a species is exceptionally shade tolerant, is markedly efficient at sunfleck use, utilizes a particular C_4_ biochemical subtype, is notably morphologically diverse, evolved in an especially low competition environment, or has circumvented difficulties in phloem loading, then it may be the exception to the rule. Only by examining all of the interconnected aspects of a complex trait such as C_4_ photosynthesis can we begin to understand why a few unique trees have travelled the pathway to C_4_, whereas other would-be C_4_ trees cannot complete the journey. New directions into understanding the rarity of C_4_ trees also merit investigation, including comparative genomic approaches and investigations into any role that the characteristic latex and laticifers of *Euphorbia* plants may play in overcoming limitations imposed by passive symplastic phloem loading and hydraulic constraints. These Hawaiian *Euphorbia* represent a crucial resource in advancing our understanding in these areas. However, they are becoming increasingly threatened in their native habitat: 10 taxa are state and federally listed as endangered and several others are observed to be rare (MJS-K, unpublished data). The narrow endemism of these species and lack of appropriate, protected habitats for conservation mean they are vulnerable to fires, invasive species, human activity, and climate change.

With Hawaiian *Euphorbia* most probably arising from a herbaceous-to-woody transition in a C_4_ ancestor (Yang and Berry, 2011), it is still unclear whether an existing tree could evolve the C_4_ trait, and there are currently no known C_3_–C_4_ intermediate tree species to indicate that this is a possibility. Indeed, the low quantum yield of these C_3_–C_4_ intermediates could mean that, at least under an existing canopy, the transition to C_4_ would require the traversal of an adaptive trough (Monson *et al.*, 1986; Stata *et al.*, 2019). Additionally, the occurrence of passive symplastic phloem loading could also act as a barrier to C_4_ evolution in existing trees. While these considerations may not apply to the evolution of a tree habit in a C_4_ ancestor, this alternative evolutionary path is still constrained by adaptive plasticity (Sage and McKown, 2006; Bellasio and Lundgren, 2016). Thus, both routes to the evolution of a C_4_ tree are potentially tortuous, which may together explain the global rarity of C_4_ tree species.

## References

[CIT0001] Bellasio C, Griffiths H 2014 Acclimation to low light by C_4_ maize: implications for bundle sheath leakiness. Plant, Cell & Environment 37, 1046–1058. 10.1111/pce.12194 24004447

[CIT0002] Bellasio C, Lundgren MR 2016 Anatomical constraints to C_4_ evolution: light harvesting capacity in the bundle sheath. New Phytologist 212, 485–496. 27375085 10.1111/nph.14063

[CIT0003] Bianconi ME, Dunning LT, Moreno-Villena JJ, Osborne CP, Christin PA 2018 Gene duplication and dosage effects during the early emergence of C_4_ photosynthesis in the grass genus *Alloteropsis*. Journal of Experimental Botany 69, 1967–1980. 29394370 10.1093/jxb/ery029 PMC6018922

[CIT0004] Burgess SS, Dawson TE 2007 Predicting the limits to tree height using statistical regressions of leaf traits. New Phytologist 174, 626–636. 17447917 10.1111/j.1469-8137.2007.02017.x

[CIT0005] Carlquist S 1970 Hawaii: a natural history. New York: Natural History Press.

[CIT0006] Chollet R, Ogren WL 1975 Regulation of photorespiration in C_3_ and C_4_ species. The Botanical Review 41, 137–179.

[CIT0007] Christin PA, Arakaki M, Osborne CP, Edwards EJ 2015 Genetic enablers underlying the clustered evolutionary origins of C_4_ photosynthesis in angiosperms. Molecular Biology and Evolution 32, 846–858. 25582594 10.1093/molbev/msu410

[CIT0008] Christin PA, Edwards EJ, Besnard G, Boxall SF, Gregory R, Kellogg EA, Hartwell J, Osborne CP 2012 *a* Adaptive evolution of C_4_ photosynthesis through recurrent lateral gene transfer. Current Biology 22, 445–449. 22342748 10.1016/j.cub.2012.01.054

[CIT0009] Christin PA, Osborne CP, Chatelet DS, Columbus JT, Besnard G, Hodkinson TR, Garrison LM, Vorontsova MS, Edwards EJ 2013 Anatomical enablers and the evolution of C_4_ photosynthesis in grasses. Proceedings of the National Academy of Sciences, USA 110, 1381–1386. 10.1073/pnas.1216777110 PMC3557070 23267116

[CIT0010] Christin PA, Osborne CP, Sage RF, Arakaki M, Edwards EJ 2011 C_4_ eudicots are not younger than C_4_ monocots. Journal of Experimental Botany 62, 3171–3181. 21393383 10.1093/jxb/err041

[CIT0011] Christin PA, Petitpierre B, Salamin N, Büchi L, Besnard G 2009 Evolution of C_4_ phosphoenolpyruvate carboxykinase in grasses, from genotype to phenotype. Molecular Biology and Evolution 26, 357–365. 18988688 10.1093/molbev/msn255

[CIT0012] Christin PA, Wallace MJ, Clayton H, Edwards EJ, Furbank RT, Hattersley PW, Sage RF, Macfarlane TD, Ludwig M 2012 *b* Multiple photosynthetic transitions, polyploidy, and lateral gene transfer in the grass subtribe Neurachninae. Journal of Experimental Botany 63, 6297–6308. 23077201 10.1093/jxb/ers282 PMC3481218

[CIT0013] Danila FR, Quick WP, White RG, Furbank RT, von Caemmerer S 2016 The metabolite pathway between bundle sheath and mesophyll: quantification of plasmodesmata in leaves of C_3_ and C_4_ monocots. The Plant Cell 28, 1461–1471. 27288224 10.1105/tpc.16.00155 PMC4944413

[CIT0014] Davidson A, Keller F, Turgeon R 2011 Phloem loading, plant growth form, and climate. Protoplasma 248, 153–163. 21125302 10.1007/s00709-010-0240-7

[CIT0015] Dunbar-Co S, Wieczorek AM, Morden CW 2008 Molecular phylogeny and adaptive radiation of the endemic Hawaiian *Plantago* species (Plantaginaceae). American Journal of Botany 95, 1177–1188. 21632435 10.3732/ajb.0800132

[CIT0016] Dunning LT, Moreno-Villena JJ, Lundgren MR, et al. 2019 *a* Key changes in gene expression identified for different stages of C_4_ evolution in *Alloteropsis semialata*. Journal of Experimental Botany 70, 3255–3268. 30949663 10.1093/jxb/erz149 PMC6598098

[CIT0017] Dunning LT, Olofsson JK, Parisod C, et al. 2019 *b* Lateral transfers of large DNA fragments spread functional genes among grasses. Proceedings of the National Academy of Sciences, USA 116, 4416–4425. 10.1073/pnas.1810031116 PMC6410850 30787193

[CIT0018] Eggens F, Popp M, Nepokroeff M, Wagner WL, Oxelman B 2007 The origin and number of introductions of the Hawaiian endemic *Silene* species (Caryophyllaceae). American Journal of Botany 94, 210–218. 21642223 10.3732/ajb.94.2.210

[CIT0019] Ehleringer J, Björkman O 1977 Quantum yields for CO_2_ uptake in C_3_ and C_4_ plants: dependence on temperature, CO_2_, and O_2_ concentration. Plant Physiology 59, 86–90. 16659794 10.1104/pp.59.1.86 PMC542335

[CIT0020] Ehleringer J, Pearcy RW 1983 Variation in quantum yield for CO_2_ uptake among C_3_ and C_4_ plants. Plant Physiology 73, 555–559. 16663257 10.1104/pp.73.3.555 PMC1066505

[CIT0021] Ehleringer JR 1978 Implications of quantum yield differences on the distributions of C_3_ and C_4_ grasses. Oecologia 31, 255–267. 28309737 10.1007/BF00346246

[CIT0022] Ehleringer JR, Cerling TE, Helliker BR 1997 C_4_ photosynthesis, atmospheric CO_2_, and climate. Oecologia 112, 285–299. 28307475 10.1007/s004420050311

[CIT0023] Ehleringer JR, Sage RF, Flanagan LB, Pearcy RW 1991 Climate change and the evolution of C_4_ photosynthesis. Trends in Ecology and Evolution 6, 95–99. 21232434 10.1016/0169-5347(91)90183-X

[CIT0024] Emms DM, Covshoff S, Hibberd JM, Kelly S 2016 Independent and parallel evolution of new genes by gene duplication in two origins of C_4_ photosynthesis provides new insight into the mechanism of phloem loading in C_4_ species. Molecular Biology and Evolution 33, 1796–1806. 27016024 10.1093/molbev/msw057 PMC4915358

[CIT0025] Givnish TJ, Millam KC, Mast AR, Paterson TB, Theim TJ, Hipp AL, Henss JM, Smith JF, Wood KR, Sytsma KJ 2009 Origin, adaptive radiation and diversification of the Hawaiian lobeliads (Asterales: Campanulaceae). Proceedings of the Royal Society B: Biological Sciences 276, 407–416. 10.1098/rspb.2008.1204 PMC2664350 18854299

[CIT0026] Griffiths H, Weller G, Toy LF, Dennis RJ 2013 You’re so vein: bundle sheath physiology, phylogeny and evolution in C_3_ and C_4_ plants. Plant, Cell & Environment 36, 249–261. 10.1111/j.1365-3040.2012.02585.x 22827921

[CIT0027] Hatch MD, Slack CR 1966 Photosynthesis by sugar-cane leaves. A new carboxylation reaction and the pathway of sugar formation. The Biochemical Journal 101, 103–111. 5971771 10.1042/bj1010103 PMC1270070

[CIT0028] Hattersley PW 1984 Characterization of C_4_ type leaf anatomy in grasses (Poaceae). mesophyll:bundle sheath area ratios. Annals of Botany 53, 163–180.

[CIT0029] Herbst D 1971 Disjunct foliar veins in hawaiian *Euphorbias*. Science 171, 1247–1248. 17742577 10.1126/science.171.3977.1247

[CIT0030] Horn JW, van Ee BW, Morawetz JJ, Riina R, Steinmann VW, Berry PE, Wurdack KJ 2012 Phylogenetics and the evolution of major structural characters in the giant genus *Euphorbia* L. (Euphorbiaceae). Molecular Phylogenetics and Evolution 63, 305–326. 22273597 10.1016/j.ympev.2011.12.022

[CIT0031] Horn JW, Xi Z, Riina R, Peirson JA, Yang Y, Dorsey BL, Berry PE, Davis CC, Wurdack KJ 2014 Evolutionary bursts in *Euphorbia* (Euphorbiaceae) are linked with photosynthetic pathway. Evolution 68, 3485–3504. 25302554 10.1111/evo.12534

[CIT0032] Kortschak HP, Hartt CE, Burr GO 1965 Carbon dioxide fixation in sugarcane leaves. Plant Physiology 40, 209–213. 16656075 10.1104/pp.40.2.209 PMC550268

[CIT0033] Koutnik DL 1987 A taxonomic revision of the Hawaiian species of the genus *Chamaesyce* (Euphorbiaceae). Allertonia 4, 331–388.

[CIT0034] Krall JP, Pearcy RW 1993 Concurrent measurements of oxygen and carbon dioxide exchange during lightflecks in maize (*Zea mays* L.). Plant Physiology 103, 823–828. 12231981 10.1104/pp.103.3.823 PMC159052

[CIT0035] Kromdijk J, Schepers HE, Albanito F, Fitton N, Carroll F, Jones MB, Finnan J, Lanigan GJ, Griffiths H 2008 Bundle sheath leakiness and light limitation during C_4_ leaf and canopy CO_2_ uptake. Plant Physiology 148, 2144–2155. 18971428 10.1104/pp.108.129890 PMC2593657

[CIT0036] Liesche J, Pace MR, Xu Q, Li Y, Chen S 2017 Height-related scaling of phloem anatomy and the evolution of sieve element end wall types in woody plants. New Phytologist 214, 245–256. 27935048 10.1111/nph.14360 PMC5347917

[CIT0037] Long SP 1999 Environmental responses. In: Sage RF, Monson RK, eds. C_4_ plant biology. Academic Press, 215–249.

[CIT0038] Lundgren MR, Besnard G, Ripley BS, et al. 2015 Photosynthetic innovation broadens the niche within a single species. Ecology Letters 18, 1021–1029. 26248677 10.1111/ele.12484

[CIT0039] Lundgren MR, Christin PA, Escobar EG, Ripley BS, Besnard G, Long CM, Hattersley PW, Ellis RP, Leegood RC, Osborne CP 2016 Evolutionary implications of C_3_–C_4_ intermediates in the grass *Alloteropsis semialata*. Plant, Cell & Environment 39, 1874–1885. 10.1111/pce.12665 26524631

[CIT0040] Lundgren MR, Dunning LT, Olofsson JK, et al. 2019 C_4_ anatomy can evolve via a single developmental change. Ecology Letters 22, 302–312. 30557904 10.1111/ele.13191 PMC6849723

[CIT0041] Lundgren MR, Osborne CP, Christin PA 2014 Deconstructing Kranz anatomy to understand C_4_ evolution. Journal of Experimental Botany 65, 3357–3369. 24799561 10.1093/jxb/eru186

[CIT0042] Monson RK 1989 On the evolutionary pathways resulting in C_4_ photosynthesis and crassulacean acid metabolism. Advances in Ecological Research 19, 57–110.

[CIT0043] Monson RK 1999 The origins of C_4_ genes and evolutionary pattern in the C_4_ metabolic phenotype. In: Sage RF, Monson RK, eds. C_4_ plant biology. Academic Press, 377–410.

[CIT0044] Monson RK 2003 Gene duplication, neofunctionalization, and the evolution of C_4_ photosynthesis. International Journal of Plant Sciences 164(3 Suppl), S43–S54.

[CIT0045] Monson RK, Moore BD 1989 On the significance of C_3_–C_4_ intermediate photosynthesis to the evolution of C_4_ photosynthesis. Plant, Cell & Environment 12, 689–699.

[CIT0046] Monson RK, Moore BD, Ku MS, Edwards GE 1986 Co-function of C_3_- and C_4_-photosynthetic pathways in C_3_, C_4_ and C_3_–C_4_ intermediate *Flaveria* species. Planta 168, 493–502. 24232325 10.1007/BF00392268

[CIT0047] Moreno-Villena JJ, Dunning LT, Osborne CP, Christin PA 2018 Highly expressed genes are preferentially co-opted for C_4_ photosynthesis. Molecular Biology and Evolution 35, 94–106. 29040657 10.1093/molbev/msx269 PMC5850498

[CIT0048] Oparka KJ, Prior DAM 1992 Direct evidence for pressure-generated closure of plasmodesmata. The Plant Journal 2, 741–750.

[CIT0049] Olofsson JK, Dunning LT, Lundgren MR, et al. 2019 Population-specific selection on standing variation generated by lateral gene transfers in a grass. Current Biology 29, 3921–3927. 31679927 10.1016/j.cub.2019.09.023

[CIT0050] Panero JL, Francisco-Ortega J, Jansen RK, Santos-Guerra A 1999 Molecular evidence for multiple origins of woodiness and a New World biogeographic connection of the Macaronesian island endemic Pericallis (Asteraceae: Senecioneae). Proceedings of the National Academy of Sciences, USA 96, 13886–13891. 10.1073/pnas.96.24.13886 PMC24160 10570168

[CIT0051] Paul MJ, Foyer CH 2001 Sink regulation of photosynthesis. Journal of Experimental Botany 52, 1383–1400. 11457898 10.1093/jexbot/52.360.1383

[CIT0052] Paul MJ, Pellny TK 2003 Carbon metabolite feedback regulation of leaf photosynthesis and development. Journal of Experimental Botany 54, 539–547. 12508065 10.1093/jxb/erg052

[CIT0053] Pearcy RW 1983 The light environment and growth of C_3_ and C_4_ tree species in the understory of a Hawaiian forest. Oecologia 58, 19–25. 28310642 10.1007/BF00384537

[CIT0054] Pearcy RW, Ehleringer JR 1984 Comparative ecophysiology of C_3_ and C_4_ plants. Plant, Cell & Environment 7, 1–13.

[CIT0055] Pearcy RW, Osteryoung K, Calkin HW 1985 Photosynthetic responses to dynamic light environments by hawaiian trees: time course of CO_2_ uptake and carbon gain during sunflecks. Plant Physiology 79, 896–902. 16664512 10.1104/pp.79.3.896 PMC1074991

[CIT0056] Pearcy RW, Troughton J 1975 C_4_ photosynthesis in tree form *Euphorbia* species from Hawaiian rainforest sites. Plant Physiology 55, 1054–1056. 16659208 10.1104/pp.55.6.1054 PMC541764

[CIT0057] Pyankov VI, Black CC, Artyusheva EG, Voznesenskaya EV, Ku MSB, Edwards GE 1999 Features of photosynthesis in *Haloxylon* species of Chenopodiaceae that are dominant plants in Central Asian Deserts. Plant & Cell Physiology 40, 125–134.

[CIT0058] Robichaux RH, Pearcy RW 1980 Photosynthetic responses of C_3_ and C_4_ species from cool shaded habitats in Hawaii. Oecologia 47, 106–109. 28309636 10.1007/BF00541783

[CIT0059] Rockwell FE, Gersony JT, Holbrook NM 2018 Where does Münch flow begin? Sucrose transport in the pre-phloem path. Current Opinion in Plant Biology 43, 101–107. 29704829 10.1016/j.pbi.2018.04.007

[CIT0060] Rondeau P, Rouch C, Besnard G 2005 NADP-malate dehydrogenase gene evolution in Andropogoneae (Poaceae): gene duplication followed by sub-functionalization. Annals of Botany 96, 1307–1314. 16243851 10.1093/aob/mci282 PMC4247081

[CIT0061] Ryan MG, Asao S 2014 Phloem transport in trees. Tree Physiology 34, 1–4. 24463392 10.1093/treephys/tpt123

[CIT0062] Sage RF 2001 *a* Environmental and evolutionary preconditions for the origin and diversification of the C_4_ photosynthetic syndrome. Plant Biology 3, 202–213.

[CIT0063] Sage RF 2001 *b* C_4_ plants. In: Levin SA, ed. Encyclopedia of biodiversity. Elsevier, 575–598.

[CIT0064] Sage RF 2014 Stopping the leaks: new insights into C_4_ photosynthesis at low light. Plant, Cell & Environment 37, 1037–1041. 10.1111/pce.12246 24818232

[CIT0065] Sage RF 2016 A portrait of the C_4_ photosynthetic family on the 50th anniversary of its discovery: species number, evolutionary lineages, and Hall of Fame. Journal of Experimental Botany 67, 4039–4056. 27053721 10.1093/jxb/erw156

[CIT0066] Sage RF, Christin PA, Edwards EJ 2011 *a* The C_4_ plant lineages of planet Earth. Journal of Experimental Botany 62, 3155–3169. 21414957 10.1093/jxb/err048

[CIT0067] Sage RF, Li M, Monson RK 1999 *a* The taxonomic distribution of C_4_ photosynthesis. In: Sage RF, Monson RK, eds. C_4_ plant biology. Academic Press, 551–584.

[CIT0068] Sage RF, McKown AD 2006 Is C_4_ photosynthesis less phenotypically plastic than C_3_ photosynthesis? Journal of Experimental Botany 57, 303–317. 16364950 10.1093/jxb/erj040

[CIT0069] Sage RF, Monson RK, Ehleringer JR, Adachi S, Pearcy RW 2018 Some like it hot: the physiological ecology of C_4_ plant evolution. Oecologia 187, 941–966. 29955992 10.1007/s00442-018-4191-6

[CIT0070] Sage TL, Sage RF, Vogan PJ, Rahman B, Johnson DC, Oakley JC, Heckel MA 2011 *b* The occurrence of C_2_ photosynthesis in *Euphorbia* subgenus *Chamaesyce* (Euphorbiaceae). Journal of Experimental Botany 62, 3183–3195. 21459765 10.1093/jxb/err059

[CIT0071] Sage RF, Sultmanis S 2016 Why are there no C_4_ forests? Journal of Plant Physiology 203, 55–68. 27481816 10.1016/j.jplph.2016.06.009

[CIT0072] Sage RF, Wedin DA, Li M 1999 *b* The biogeography of C_4_ photosynthesis: patterns and controlling factors. In: Sage RF, Monson RK, eds. C_4_ plant biology. Academic Press, 313–373.

[CIT0073] Savage JA, Beecher SD, Clerx L, Gersony JT, Knoblauch J, Losada JM, Jensen KH, Knoblauch M, Holbrook NM 2017 Maintenance of carbohydrate transport in tall trees. Nature Plants 3, 965–972. 29209083 10.1038/s41477-017-0064-y

[CIT0074] Schüssler C, Freitag H, Koteyeva N, Schmidt D, Edwards G, Voznesenskaya E, Kadereit G 2017 Molecular phylogeny and forms of photosynthesis in tribe Salsoleae (Chenopodiaceae). Journal of Experimental Botany 68, 207–223. 28003310 10.1093/jxb/erw432 PMC5853613

[CIT0075] Sowiński P, Bilska A, Barańska K, Fronk J, Kobus P 2007 Plasmodesmata density in vascular bundles in leaves of C_4_ grasses grown at different light conditions in respect to photosynthesis and photosynthate export efficiency. Environmental and Experimental Botany 61, 74–84.

[CIT0076] Sowiński P, Szczepanik J, Minchin PE 2008 On the mechanism of C_4_ photosynthesis intermediate exchange between Kranz mesophyll and bundle sheath cells in grasses. Journal of Experimental Botany 59, 1137–1147. 18375930 10.1093/jxb/ern054

[CIT0077] Sporck MJ 2011 The Hawaiian C_4_ Euphorbia adaptive radiation: an ecophysiological approach to understanding leaf trait diversification. PhD thesis, University of Hawaii, Manoa.

[CIT0078] Stata M, Sage TL, Hoffmann N, Covshoff S, Ka-Shu Wong G, Sage RF 2016 Mesophyll chloroplast investment in C_3_, C_4_ and C_2_ species of the genus *Flaveria*. Plant & Cell Physiology 57, 904–918. 26985020 10.1093/pcp/pcw015

[CIT0079] Stata M, Sage TL, Sage RF 2019 Mind the gap: the evolutionary engagement of the C_4_ metabolic cycle in support of net carbon assimilation. Current Opinion in Plant Biology 49, 27–34. 31150949 10.1016/j.pbi.2019.04.008

[CIT0080] Tcherkez GG, Farquhar GD, Andrews TJ 2006 Despite slow catalysis and confused substrate specificity, all ribulose bisphosphate carboxylases may be nearly perfectly optimized. Proceedings of the National Academy of Sciences, USA 103, 7246–7251. 10.1073/pnas.0600605103 PMC1464328 16641091

[CIT0081] Turgeon R 2010 The role of phloem loading reconsidered. Plant Physiology 152, 1817–1823. 20200065 10.1104/pp.110.153023 PMC2850027

[CIT0082] Yang Y 2012 Phylogenetics and evolution of Euphorbia Subgenus Chamaesyce. PhD thesis, University of Michigan, Ann Arbor.

[CIT0083] Yang Y, Berry PE 2011 Phylogenetics of the *Chamaesyce* clade (*Euphorbia*, Euphorbiaceae): reticulate evolution and long-distance dispersal in a prominent C_4_ lineage. American Journal of Botany 98, 1486–1503. 21875975 10.3732/ajb.1000496

[CIT0084] Yang Y, Morden CW, Sporck-Koehler MJ, Sack L, Wagner WL, Berry PE 2018 Repeated range expansion and niche shift in a volcanic hotspot archipelago: radiation of C_4_ Hawaiian *Euphorbia* subgenus *Chamaesyce* (Euphorbiaceae). Ecology and Evolution 8, 8523–8536. 30250720 10.1002/ece3.4354 PMC6145001

[CIT0085] Zimmermann NFA, Ritz CM, Hellwig FH 2010 Further support for the phylogenetic relationships within *Euphorbia* L. (Euphorbiaceae) from nrITS and trnL-trnF IGS sequence data. Plant Systematics and Evolution 286, 39–58.

[CIT0086] Zwieniecki MA, Boyce CK, Holbrook NM 2004 Hydraulic limitations imposed by crown placement determine final size and shape of *Quercus rubra* L. leaves. Plant, Cell and Environment 27, 357–365.

